# Supplementary material for: The predicted stem-loop structure in the 3′-end of the human norovirus antigenomic sequence is required for its genomic RNA synthesis by its RdRp
Source: J Biol Chem. 2021 Sep 23;297(4):101225. doi: 10.1016/j.jbc.2021.101225 (PMC8526979; doi:10.1016/j.jbc.2021.101225)
Supplement: Suppleemntal Figures S1–S5 and Table [file mmc1.pdf]

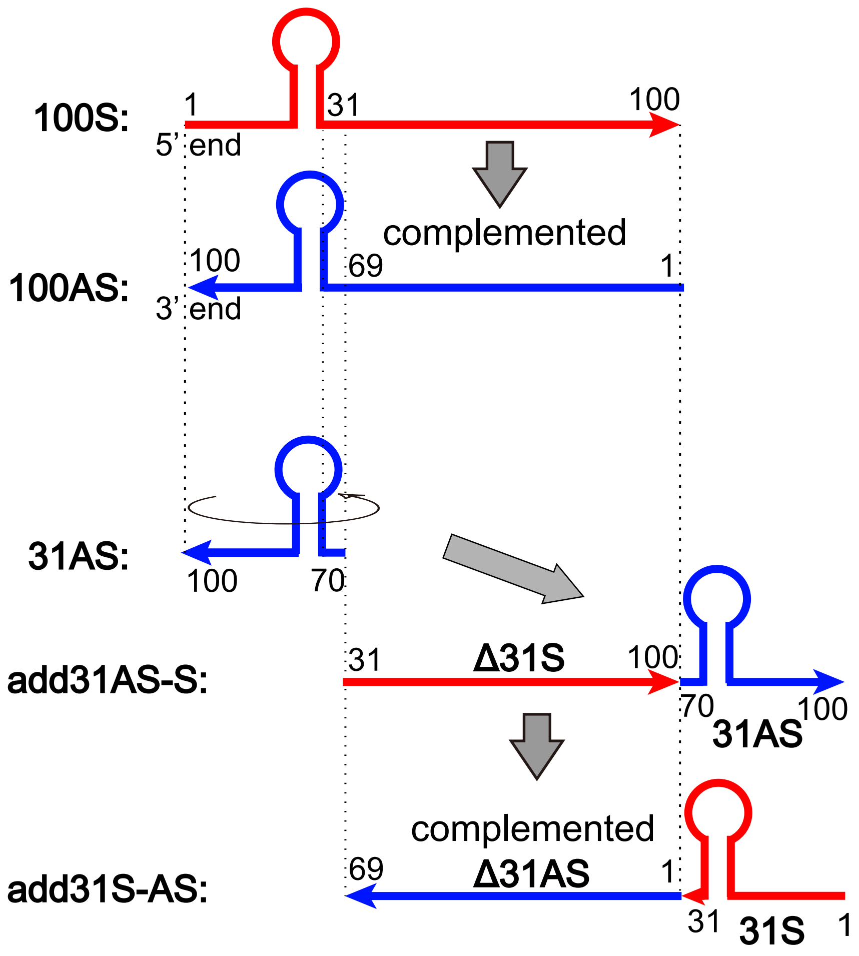

Figure S1. The schematic constructing procedures and those structures of the template add31AS-S and add31S-AS, respectively. The schematic structure of the 31AS RNA is also shown.

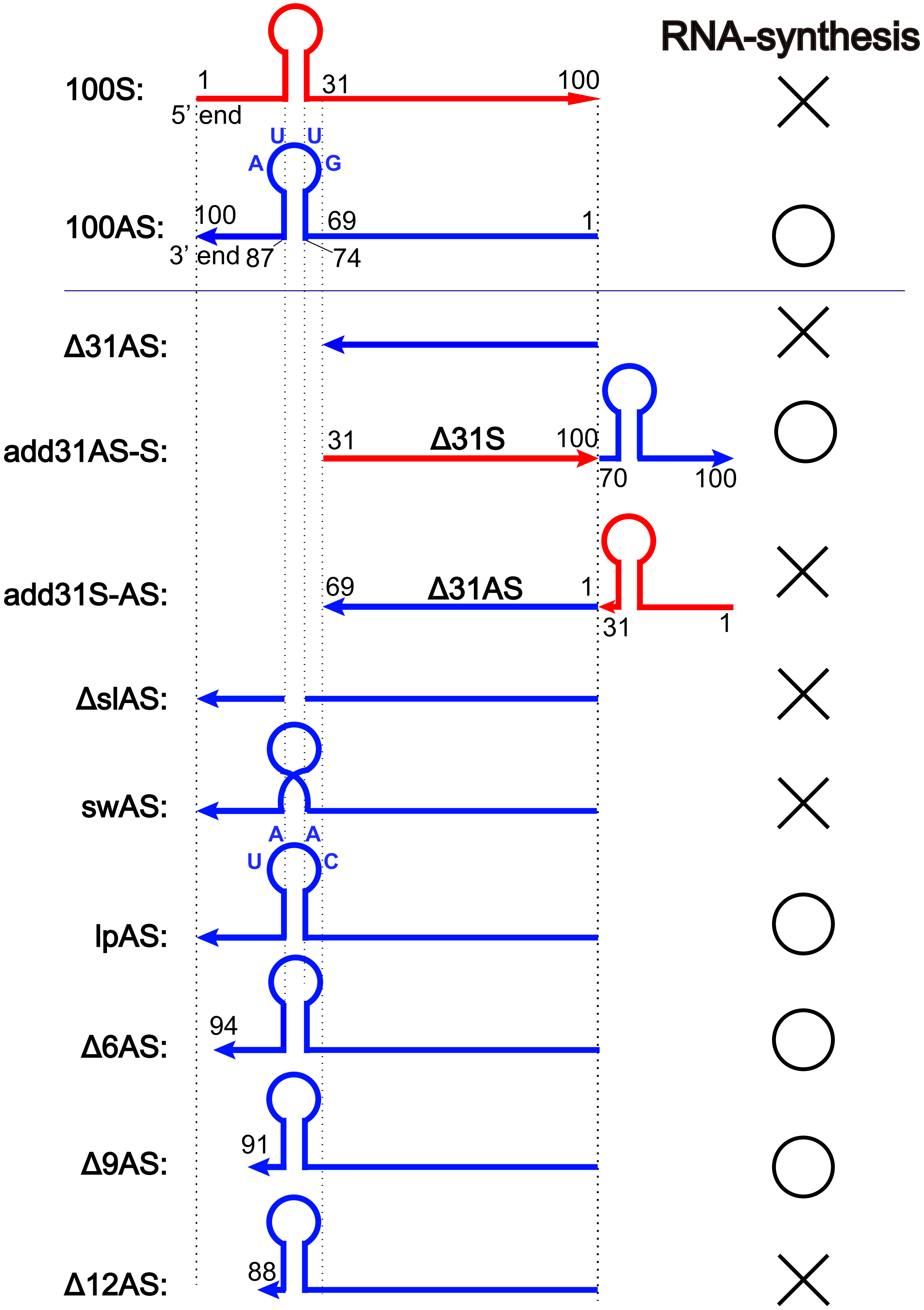

RNA-synthesis
100S:
1
31
100
5' end
U
U
A
G
100AS:
100
69
1
3' end
87
74
Δ31AS:
add31AS-S:
31
Δ31S
100
70
100
add31S-AS:
69
Δ31AS
1
31
1
ΔslAS:
swAS:
A
A
U
C
lpAS:
94
Δ6AS:
91
Δ9AS:
88
Δ12AS:

Fig. S2

Figure S2. Summary of the activities of 100S, 100AS, and its mutated template RNAs

The schematic notation of the original and mutated template 100S and 100AS RNAs are indicated. The red and blue lines indicate the sense and antisense viral genome RNA, respectively. The activities of RNA synthesis using each template RNA are shown at the right side of each template RNA with circles (active as template) and crosses (inactive as template).

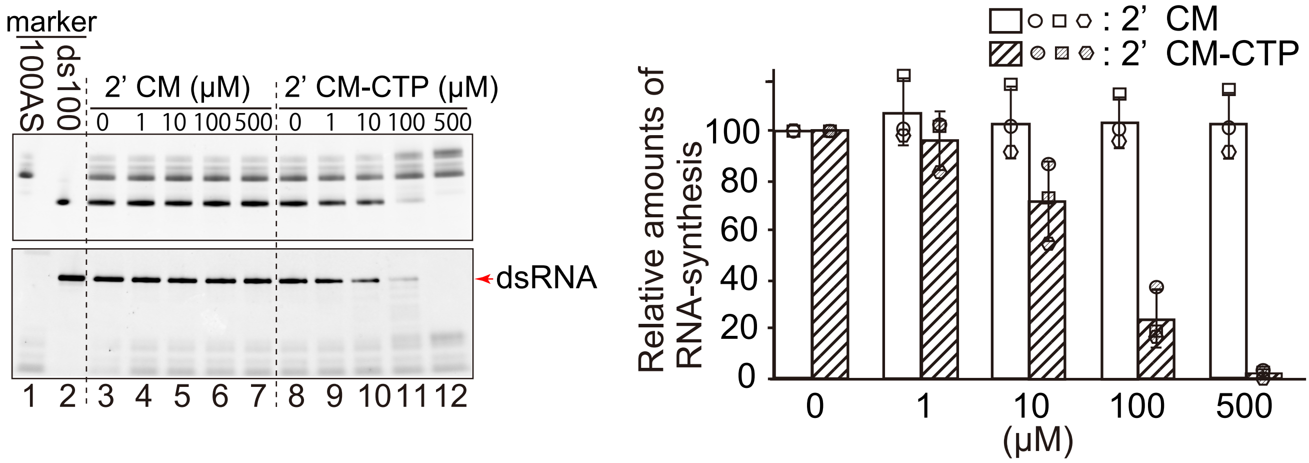

Figure S3. Inhibitory effect of 2′ CM-CTP on the RNA synthesis by RdRp

2′-C-methylcytidine (2′ CM; 0–500 μM; lanes 3–7) or 2′-C-methylcytidine triphosphate (2′ CM-CTP; 0–500 μM; lanes 8–12) was mixed with 100AS RNA (10 pmol, final concentration: 0.4 μM) and the RdRp (45 pmol, final concentration: 1.8 μM). Half of the samples were loaded on native PAGE (the upper column). The other half of the samples were treated with S1 ribonucleases and loaded on native PAGE (the lower column). The template 100AS RNA (lane 1), and ds100 RNA (lane 2) were used as the size markers. Red arrow indicates the dsRNA products. The relative amount of dsRNA products is shown in the graph. The amounts were the averages of three times experiments. The standard deviations are also shown in the graph. Each result of the three independent experiments is also shown by the three kinds of shapes (circles, rectangles, and hexagons).

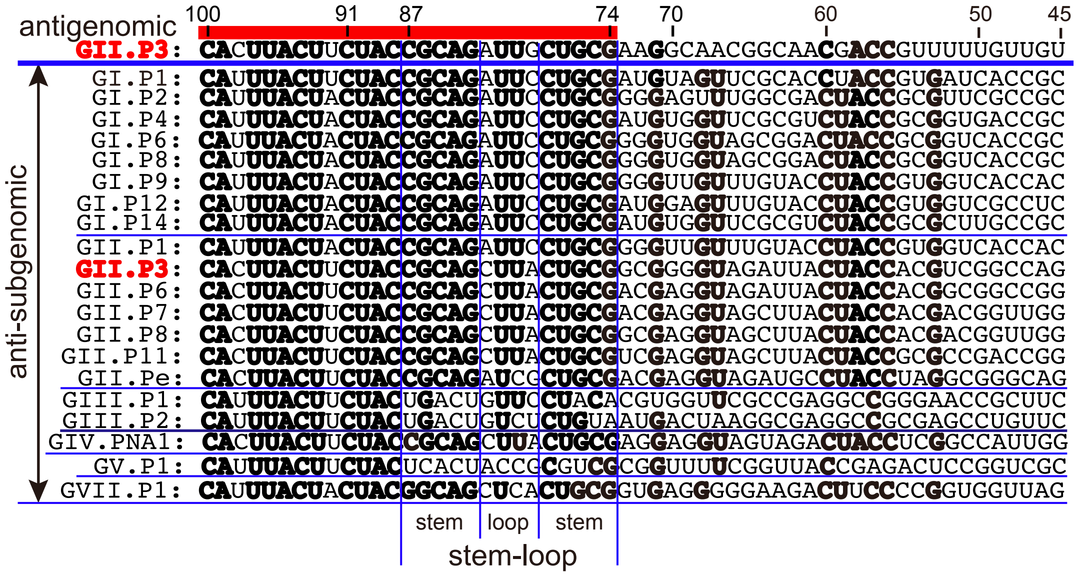

Figure S4. Comparison of the 3′ -end regions between the antisense genomic and the antisense subgenomic RNAs

Alignments of 20 kinds of norovirus genotypes at the 3 ′ -end regions of the antisense subgenomic sequences are compared with those of the antisense genomic RNA (nt 100–45 of 100AS RNA of the GII. P3 U201 strain; shown at the top). The nucleotides with more than 71% identity (the same nucleotide in more than 15 of the total 21 genotypes, including the antisense genomic GII.P3 U201 RNA) are indicated in bold. RdRp-coding genes are used to classify the genotypes (24). Horizontal blue lines separate genogroups. The red line on the top indicates highly conserved regions (nt 100–74 regions of the GII.P3 U201 strain). Four vertical blue lines show the separation in the stem and loop regions in the predicted stem-loop regions. The GII. P3 U201 strain that was used in this paper is shown in red characters. The antisense subgenomic sequences of GVI were not aligned because no subgenomic sequence data are available.

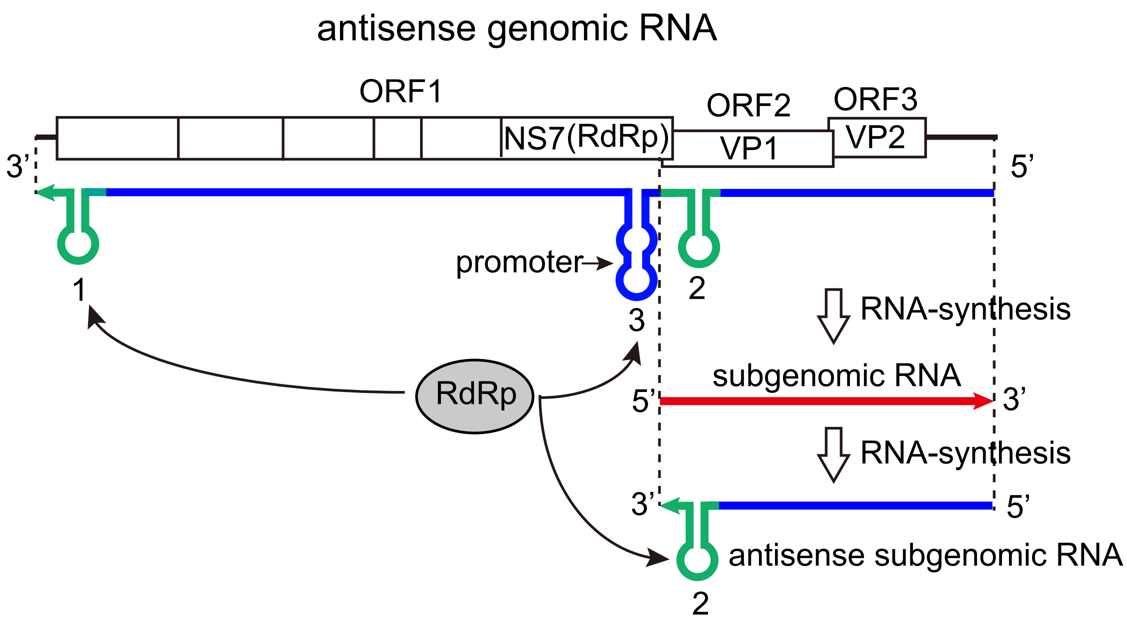

Figure. S5. Model for norovirus RdRp recognition of three regions of stem-loop structures in the antisense genomic RNA.

There are three possible regions for norovirus RdRp binding; region 1 is located at the 3′-end region of the antisense genomic RNA shown in this report. Region 2 is located at the 3 ′ -end region of the antisense subgenomic RNA, which is predicted based on high sequence similarity (Fig. S4). Region 3 is the promoter reported previously (10,13,14). When the RdRp binds to region 1 (indicated by the arrow to region 1), this initiates the RNA synthesis of the genomic RNA. When the RdRp binds to regions 2 or 3, it initiates the synthesis of the same subgenomic RNA. The RdRp binds to the promoter in the antisense genomic RNA preferentially (indicated by the arrow to the region 3), but once region 2 becomes the 3′ -end, the RdRp binds to region 2 preferentially (indicated by the arrow to region 2).

Table; Primers for plasmid-preparation of RdRp and template RNAs

name of plasmid	name of primer	Sequenses
pET RdRp	U201RdRp S	GGAATTCCATATGGGAGGTGACGACAAGGGCAC
	U201RdRp AS	GGAATTCTCATTCGACGCCATCTTCAT
pUC 100S	1S S	GCTCTAGATAATACGACTCACTATAGGGTGAATGAAGATGGCGTCTA
	1S AS	GGAATTCGGTCTCTGGGTTAGAAAACAAGCTGTCAC
pUC 100AS	1AS S	GCTCTAGATAATACGACTCACTATAGGGTTAGAAAACAAGCTGTCAC
	1AS AS	GGAATTCGGTCTCTGGGTGAATGAAGATGGCGTCTA
pUC d31S	1S 32S	GCTCTAGATAATACGACTCACTATAGGGTTGCCGTTGCTGGCAAAAA
	1S AS	GGAATTCGGTCTCTGGGTTAGAAAACAAGCTGTCAC
pUC d31AS	1A S	GCTCTAGATAATACGACTCACTATAGGGTTAGAAAACAAGCTGTCAC
	1AS-32AS	GGAATTCGGTCTCTGGGTTGCCGTTGCTGGCAAAAA
pUC dslS	noSLS	GCTCTAGATAATACGACTCACTATAGGGTGAATGAAGATGGCTTCCGTTGC
	1S AS	GGAATTCGGTCTCTGGGTTAGAAAACAAGCTGTCAC
pUC dslAS	1A S	GCTCTAGATAATACGACTCACTATAGGGTTAGAAAACAAGCTGTCAC
	noSLAS	GCTCTAGATAATACGACTCACTATAGGGTTAGAAAACAAGCTGTCAC
pUC swS	1sw	GCTCTAGATAATACGACTCACTATAGGGTGAATGAAGATGGCTTCCGTTGC
	1S AS	GGAATTCGGTCTCTGGGTTAGAAAACAAGCTGTCAC
pUC swAS	1A S	GCTCTAGATAATACGACTCACTATAGGGTTAGAAAACAAGCTGTCAC
	swAS	GGAATTCGGTCTCTGGGTGAATGAAGATGGGCAGCAATCTGC CTTCCGTTGC
pUC slS	XbaT7loopATTG S	GCTCTAGATAATACGACTCACTATAGGGTGAATGAAGATGGCGTCATTGGACGCTTCC
	1S-AS	GGAATTCGGTCTCTGGGTTAGAAAACAAGCTGTCAC
pUC slAS	1A S	GCTCTAGATAATACGACTCACTATAGGGTTAGAAAACAAGCTGTCAC
	loopATTG AS	GGAATTCGGTCTCTGGGTGAATGAAGATGGCGTCATTGGACGCTTCC
pUC d6S	Xba d1-6S	GCTCTAGATAATACGACTCACTATAGGGAAGATGGCGTCTAACGACG
	1S AS	GGAATTCGGTCTCTGGGTTAGAAAACAAGCTGTCAC
pUC d6AS	1A S	GCTCTAGATAATACGACTCACTATAGGGTTAGAAAACAAGCTGTCAC
	d6 AS	GGAATTCGGTCTCTGGGAAGATGGCGTCTAACGACG
pUC d9S	Xba d1-9S	GCTCTAGATAATACGACTCACTATAGGGATGGCGTCTAACGACGCTT
	1S AS	GGAATTCGGTCTCTGGGTTAGAAAACAAGCTGTCAC
pUC d9AS	1A S	GCTCTAGATAATACGACTCACTATAGGGTTAGAAAACAAGCTGTCAC
	d9 AS	GGAATTCGGTCTCTGGGATGGCGTCTAACGACGCTT
pUC d12S	Xba d1-12S	GCTCTAGATAATACGACTCACTATAGGGCGTCTAACGACGCTTCCG
	1S AS	GGAATTCGGTCTCTGGGTTAGAAAACAAGCTGTCAC
pUC d12AS	1A S	GCTCTAGATAATACGACTCACTATAGGGTTAGAAAACAAGCTGTCAC
	d12 AS	GGAATTCGGTCTCTGGGCGTCTAACGACGCTTCCG
pUC add31AS S	1S 32 S	GCTCTAGATAATACGACTCACTATAGGGTTGCCGTTGCTGGCAAAAA
	100add31S-AS	GGAATTCGGTCTCTGGGTGAATGAAGATGGCGTCTAACGACGCTTCCGTTAGAAAACAAGCTGTCAC
pUC add31S AS	100add31AS-S	GCTCTAGATAATACGACTCACTATAGGGTGAATGAAGATGGCGTCTAACGACGCTTCCGTTAGAAAACAAGCTGTCAC
	1AS 32AS	GGAATTCGGTCTCTGGGTTGCCGTTGCTGGCAAAAA
